# Supplementary material for: Crescents formations are independently associated with higher mortality in biopsy-confirmed immunoglobulin A nephropathy
Source: PLoS One. 2020 Jul 31;15(7):e0237075. doi: 10.1371/journal.pone.0237075 (PMC7394392; doi:10.1371/journal.pone.0237075)
Supplement: S1 Table — (DOCX) [file pone.0237075.s001.docx]

**S1 Table. All patients with crescent IgAN.**

| Case number | 45 |
| --- | --- |
| Mean | 23% |
| Median | 18% |
| Standard deviation | 18.85% |
| Maximal | 88% |
| Minimal | 2% |
