# Supplementary material for: Crescents formations are independently associated with higher mortality in biopsy-confirmed immunoglobulin A nephropathy
Source: PLoS One. 2020 Jul 31;15(7):e0237075. doi: 10.1371/journal.pone.0237075 (PMC7394392; doi:10.1371/journal.pone.0237075)
Supplement: S2 Table — (DOCX) [file pone.0237075.s002.docx]

**S2 Table. Distribution of the proportions of crescents.**

| Percentage of crescent involvement | <5% | 5-9% | 10-25% | ≥ 25% | All crescent |
| --- | --- | --- | --- | --- | --- |
| Case number | 3 | 8 | 17 | 17 | 45 |
| Percentage (%) | 6.7 | 17.8 | 37.8 | 37.8 | 100 |
