# Supplementary material for: Crescents formations are independently associated with higher mortality in biopsy-confirmed immunoglobulin A nephropathy
Source: PLoS One. 2020 Jul 31;15(7):e0237075. doi: 10.1371/journal.pone.0237075 (PMC7394392; doi:10.1371/journal.pone.0237075)
Supplement: S3 Table — (DOCX) [file pone.0237075.s003.docx]

**S3 Table. Cause of death.**

|  | Infection related | Non-infection related |
| --- | --- | --- |
| No crescent IgAN (8) | Pneumonia, lung abscess, pneumonia*3: 62.5% | Hepatoma, tension pneumothorax, gastrointestinal bleeding: 37.5% |
| Crescent IgAN (4) | Bacteremia*2, acute cholecystitis: 75% | Arrhythmia: 25% |
