# Supplementary figures and images for: Crescents formations are independently associated with higher mortality in biopsy-confirmed immunoglobulin A nephropathy
Source: PLoS One. 2020 Jul 31;15(7):e0237075. doi: 10.1371/journal.pone.0237075 (PMC7394392; doi:10.1371/journal.pone.0237075)

**S1 Fig. Distribution of the proportions of glomeruli with crescents.**
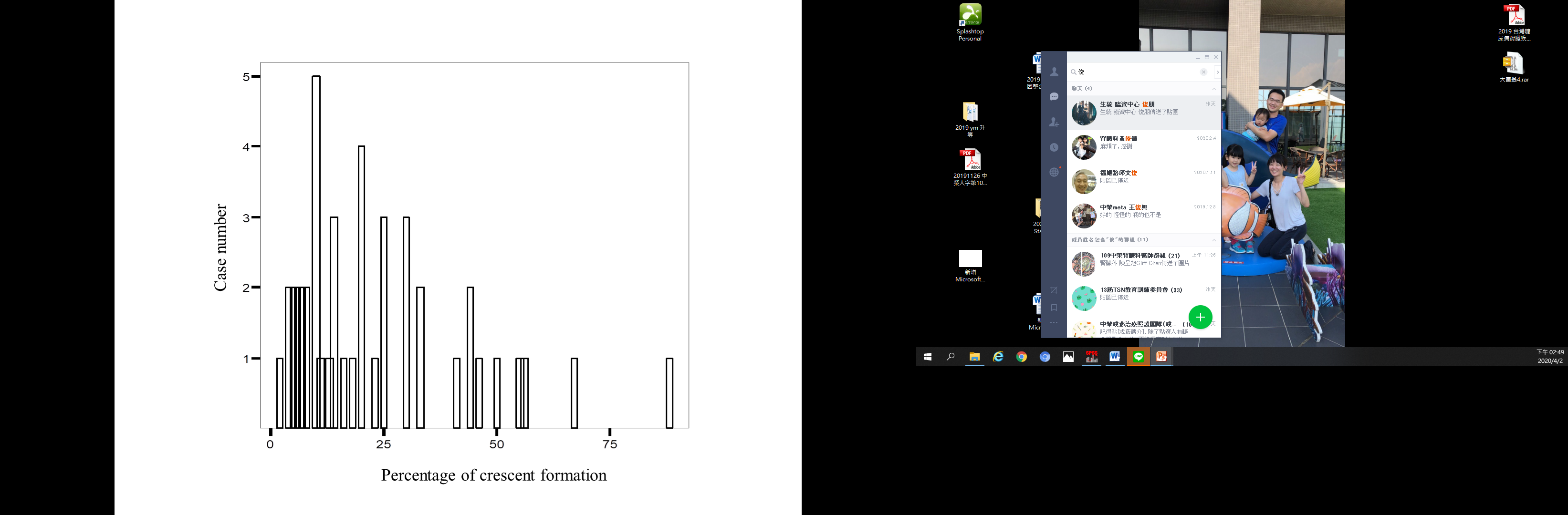

Supplement: S1 Fig — (DOCX) [file pone.0237075.s004.docx]
