# Supplementary material for: Crescents formations are independently associated with higher mortality in biopsy-confirmed immunoglobulin A nephropathy
Source: PLoS One. 2020 Jul 31;15(7):e0237075. doi: 10.1371/journal.pone.0237075 (PMC7394392; doi:10.1371/journal.pone.0237075)
Supplement: Attachment — Submitted filename: response to reviewer comment.docx [file pone.0237075.s006.docx]

Reviewer #1: This referee invite the authors to revise the manuscript according to the suggestions listed in the first comments, otherwise the paper will be rejected by this referee.
Kidney biopsies must be revised according to the Oxford classification described by Haas et al in JASN 28:691-701, 2017.

We reviewed our data regarding renal biopsy, and we re-scored it by Oxford classification with MEST-C score. We replaced Haas classification with Oxford classification in table 1. We also revised some parts in text. Thanks for your suggestions.

|  | Total (n=388) | | Non Crescents(n=343) | | Crescents (n=45) | | *p* value |
| --- | --- | --- | --- | --- | --- | --- | --- |
| Oxford classification (n, %) |  |  |  |  |  |  |  |
| M0 |  | 247 | 223 | (90.3%) |  | 24 (53.3%) | 0.078 |
| M1 |  | 138 | 118 | (34.4%) |  | 20 (44.4%) | 0.087 |
| M2 |  | 3 | 2 | (0.6%) | 1 | (2.2%) | 0.227 |
| E0 |  | 227 | 296 | (86.3%) | 31 | (68.9%) | 0.04 |
| E1 |  | 62 | 48 | (14.0%) | 14 | (31.1%) | 0.002 |
| S0 |  | 341 | 322 | (93.9%) | 19 | (45.2%) | <0.001 |
| S1 |  | 47 | 21 | (6.1%) | 26 | (57.8%) | <0.001 |
| T0 |  | 332 | 298 | (86.9%) | 34 | (75.6%) | 0.073 |
| T1 |  | 38 | 29 | (8.5%) | 9 | (20%) | 0.028 |
| T2 |  | 18 | 16 | (4.7%) | 2 | (4.4%) | 0.788 |
| C0 |  | 388 | 343 | (100%) | 0 | (0%) | <0.001 |
| C1 |  | 35 | 0 |  | 35 | (77.8%) | <0.001 |
| C2 |  | 10 | 0 |  | 10 | (22.2%) | <0.001 |
